# Biochemical characterization of bovine plasma thrombin-activatable fibrinolysis inhibitor (TAFI)

**DOI:** 10.1186/1471-2091-10-13

**Published:** 2009-05-05

**Authors:** Zuzana Valnickova, Morten Thaysen-Andersen, Peter Højrup, Trine Christensen, Kristian W Sanggaard, Torsten Kristensen, Jan J Enghild

**Affiliations:** 1 From Center for Insoluble Protein Structures (inSPIN) and Interdisciplinary Nanoscience Center (iNANO), Department of Molecular Biology, Science Park, University of Aarhus, Gustav Wieds Vej 10c, 8000 Aarhus C, Denmark; 2 Department of Biochemistry and Molecular Biology, University of Southern Denmark, Campusvej 55, DK-5230 Odense M, Denmark

## Abstract

**Background:**

TAFI is a plasma protein assumed to be an important link between coagulation and fibrinolysis. The three-dimensional crystal structures of authentic mature bovine TAFI (TAFIa) in complex with tick carboxypeptidase inhibitor, authentic full lenght bovine plasma thrombin-activatable fibrinolysis inhibitor (TAFI), and recombinant human TAFI have recently been solved. In light of these recent advances, we have characterized authentic bovine TAFI biochemically and compared it to human TAFI.

**Results:**

The four N-linked glycosylation sequons within the activation peptide were all occupied in bovine TAFI, similar to human TAFI, while the sequon located within the enzyme moiety of the bovine protein was non-glycosylated. The enzymatic stability and the kinetic constants of TAFIa differed somewhat between the two proteins, as did the isoelectric point of TAFI, but not TAFIa. Equivalent to human TAFI, bovine TAFI was a substrate for transglutaminases and could be proteolytically cleaved by trypsin or thrombin/solulin complex, although small differences in the fragmentation patterns were observed. Furthermore, bovine TAFI exhibited intrinsic activity and TAFIa attenuated tPA-mediated fibrinolysis similar to the human protein.

**Conclusion:**

The findings presented here suggest that the properties of these two orthologous proteins are similar and that conclusions reached using the bovine TAFI may be extrapolated to the human protein.

## Background

Human thrombin-activatable fibrinolysis inhibitor (TAFI) (EC 3.4.17.20; UniProt, Q96IY4), also known as plasma pro-carboxypeptidase B, R, and U, is a plasma metallocarboxypeptidase that attenuates fibrinolysis [1-10]. TAFI circulates in plasma as a 58 kDa protein with significant intrinsic activity [11,12]. The majority of the sites that undergo transglutaminase-mediated cross-linking to fibrin are primarily located on the heavily glycosylated pro-peptide, suggesting that TAFI becomes incorporated into the fibrin clot during later stages of the coagulation cascade [13]. A variety of trypsin-like proteinases have been shown to remove this peptide, generating the mature protein, TAFIa [4,14-17]. The isoelectric point (pI) of this proteolytically cleaved protein is around pH 8.5, which is significantly more basic than that of TAFI (pI 5.5) [18]. TAFIa remains in circulation by forming complexes with α_2_-macroglobulin and pregnancy zone protein [19] but is highly unstable, a feature initially attributed to proteolytic cleavage. However, this instability is now thought to result from a temperature-dependent conformational change that occurs within minutes of activation [4,20-22].

TAFI has been implicated not only in fibrinolysis, but also in inflammation, wound healing, and a variety of other deficiencies and diseases, such as diabetes, kidney failure, lung cancer, and liver illnesses [23-29]. Interestingly, individuals with the more stable Ile^325 ^variant are apparently more susceptible to meningococcal sepsis [30]. TAFI has been studied in multiple animal models, including dog, rabbit, mouse, and rat [31-36]. Intriguingly, the absence of the protein in knock out mice is compatible with murine life [25,37,38].

Mouse and rat TAFI have been characterized, and both show similarity to the human protein [32,33,35]. Until very recently, the only available structural model for the study of TAFI was human pancreatic pro carboxypeptidase B (pro-CPB) [39]. The protein sequence of Pro-CPB is about 40% identical to TAFI. However, in contrast to TAFI, pro-CPB lacks intrinsic activity and its active form, carboxypeptidase B (CPB), is stable upon activation [40]. Efforts to crystallize authentic human TAFI have been unsuccessful, most likely due to its sugar heterogeneity when purified from pooled plasma [18]. However, using recombinant human TAFI and authentic protein purified from a single cow enabled the zymogen structure to be solved [41,42]. Although bovine TAFI is similar to pro-CPB, it also has differences. Significantly, the position of the pro-peptide is rotated 12° away from the active site, exposing access to the catalytic residues. Another significant distinction is the lack of the corresponding salt bridge between Asp^41 ^and Arg^145 ^in TAFI [42]. These distinctions might explain the intrinsic activity of TAFI [11,12]. Furthermore, the structure of bovine TAFIa in complex with tick carboxypeptidase inhibitor (TCI) was determined and found to exhibit a high degree of identify with the CPB-TCI structure [43-45]. Interestingly, the bovine TAFIa structure contains two undefined regions, both of which are part of exposed loops present in the Lβ2β3 and Lα2β4 regions and in a heparin affinity region [45]. The domains including Arg^302 ^and Arg^330^, which are predicted to cause instability in human TAFI, were fully ordered in the bovine molecule.

These recent advances prompted us to perform a thorough biochemical characterization of the bovine protein, purified from bovine plasma. This biochemical characterization included analysis of stability, N-linked glycosylation, generation of TAFIa by removal of the pro-peptide by trypsin and thrombin/solulin, the antifibrinolytic effects of TAFIa, as well as analysis of the intrinsic activity of the full length protein and its potential to become crosslinked to fibrin by transglutaminases.

## Results

### Primary structure of bovine TAFI

The amino acid sequence of bovine TAFI was deduced from a cDNA library and published recently [45]. The sequence was 78.6% identical to that of the human protein. The bovine protein consisted of 401 amino acid residues, including a 92-amino acid residue pro-peptide that is released by cleavage at Arg^92^. All potential glycosylation sites were conserved and found glycosylated in both species, with exception of the fifth site (Asn^219^), which remained unglycosylated in bovine TAFI. The location of cysteine residues was identical in both species, with the exception of Cys^69^. This cysteine residue, which is located in the activation peptide, was absent from bovine TAFI. In human TAFI, Cys^69 ^does not form a disulfide bridge and therefore, is unlikely to affect tertiary structure. All sites involved in catalysis as well as substrate and zinc binding were identical, suggesting that the two proteins have the same proteolytic properties.

### Generation and activity of TAFIa

**Figure 1:**
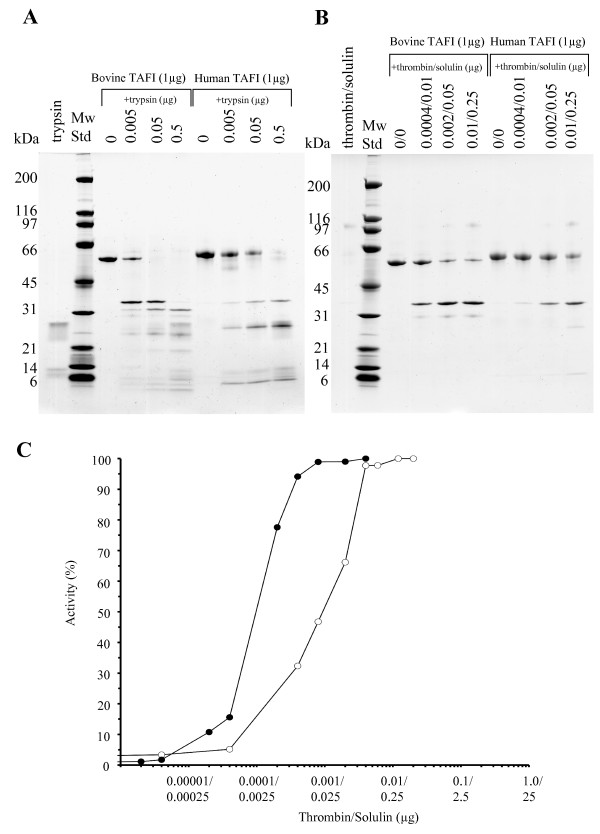
**Generation of bovine and human TAFIa**. Bovine and human TAFI (1 μg) were incubated with increasing amounts of trypsin (A) or thrombin/solulin complex (B) (all values in μg). Proteolysis products were then analyzed by SDS-PAGE and visualized by Coomassie Brilliant Blue staining. Additionally, TAFI (0.2 μg of bovine or human) was incubated with increasing amounts of thrombin/solulin complex (C). Increase in activity of bovine (filled circles) and human (open circles) TAFIa was monitored through HPLC based kinetic assay using Hip-Arg substrate as described in the method section. Note that compared to human TAFI, roughly 15 times less proteinase complex is required to generate 100% active bovine TAFIa.

SDS-PAGE of purified bovine TAFI produced a single sharp band at around 56 kDa, which is slightly lower than the position of human TAFI (Fig 1). TAFIa generated by either trypsin (Fig 1A) or thrombin/solulin complex (Fig 1B) migrated at the same position for both species, suggesting that differences in the migration of the full-length species can be attributed to differences in carbohydrates attached to the pro-peptide. It is obvious from the results of the SDS-PAGE that greater amounts of proteinases were required to generate human TAFIa (Fig 1). Since the thrombin/solulin complex is considered to be responsible for release of the TAFI pro-peptide *in vivo *[14] and since trypsin seems to inactivate TAFIa more aggressively, only the complex was used to determine the optimal conditions for generation of bovine and human TAFIa. Increasing amounts of the thrombin/solulin were incubated with TAFI, and the enhanced activity was monitored by HPLC based activity assay. As shown in Fig 1C, the amount of proteinase complex required to achieve 100% TAFIa activity was much lower than that needed for the human TAFI. Furthermore, the kinetic constants for both species were determined (Table 1). The intrinsic activity of TAFI is similar between species, while the Vmax and Km for bovine TAFa is somewhat higher in comparison to the human protein.

**Table 1:** Summary of bovine and human TAFI and TAFIa kinetic values

	**Human TAFI**		**Bovine TAFI**		**Human TAFIa**		**Bovine TAFIa**	
	
Equation*	**Vmax**	**Km**	**Vmax**	**Km**	**Vmax**	**Km**	**Vmax**	**Km**
Hanes	40.17	3.41	31.67	4.63	106.00	3.14	330.67	7.86

Eadie-Hofstee	43.00	3.96	32.50	4.65	113.33	3.68	332.00	7.84

Eisenthal-Cornish-Bowden	40.83	3.91	33.33	4.69	112.67	3.74	329.33	8.24

Hyperbolic Regression	41.33 ± 7.93	3.70 ± 1.51	33.40 ± 6.33	4.85 ± 2.50	106.67 ± 8.34	2.83 ± 0.89	326.67 ± 39.67	5.28 ± 1.96

Average values	41.33	3.75	32.73	4.71	109.67	3.35	329.67	7.31

Kcat (min^-1^)	243.12		192.53		4386.80		13186.80	

Kcat/Km (min^-1^/mM)	64.83		40.88		1309.49		1803.94	

*The values for Km and Vmax were determined by the direct fit of the Michaelis-Menten equation employing four graphical methods. The data represent the enzyme-catalysed reaction for 0.33 μM TAFI and 0.025 μM TAFIa.

### Identification of proteolysis products generated upon proteinase addition to TAFI

**Figure 2:**
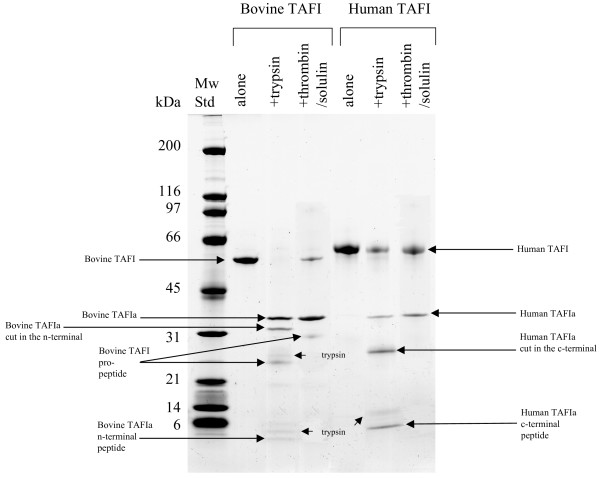
**Identification of bovine TAFI products generated by proteolysis**. SDS-PAGE of bovine or human TAFI (1 μg), which was cleaved using either 0.05 μg of trypsin, or thrombin/solulin complex in a ratio of 0.002 μg/0.05 μg. The products are indicated with arrows and were identified by Edman-degradation (see Table 2 for a summary of the bovine TAFI products).

The thrombin/solulin complex produced similar proteolytic fragmentation in both the bovine and human TAFI. The generated products were identified by Edman degradation and are summarized in Table 2. SDS-PAGE of bovine TAFI and thrombin/solulin mixture fashioned a strong band not only at 56 kDa (corresponding to full length TAFI), but also at 36 kDa, which was confirmed to be TAFIa (Fig 2 and Table 2). In contrast to the human TAFI pro-peptide, the released bovine pro-peptide was clearly visible by Coomassie staining, with a mass of around 29 kDa (Fig 2). As expected, large amounts of thrombin/solulin complex truncated human TAFIa (36 kDa) at Arg^302^, liberating the 11.0 kDa C-terminal peptide to produce a proteolytically inactivated form of TAFIa (24.7 kDa) (Fig 1B). However, no further proteolytic products were observed for the bovine protein in the higher end of the titration using the proteinase complex.

**Table 2:** Summary of bovine TAFI activation products

Product**	**Proteinase used** **in activation**	**SDS-PAGE** **mass (kDa)**	**Theoretical mass (kDa)**	N-terminal sequence*
Bovine TAFI zymogen	N/A (full length TAFI)	56	46.4	*FQRGVLSALP*

Bovine TAFIa	trypsin solulin/thrombin	36	35.9	*ASSSYYEQYH*

Bovine TAFIa cut at the n-terminus	trypsin	32	29.2	*AKNAMWID*

Bovine TAFI activation peptide	trypsin solulin/thrombin	29	10.5	*FQRGVLSALP*

Bovine TAFI activation peptide	trypsin	25	*NA*	*FQRGVLSALP*

Bovine TAFIa N-terminal peptide	trypsin	<6	6.7	*ASSSYYEQYH*

*Determined by Edman Degradation.

**Upon cleavage of bovine TAFI by either trypsin or solulin/thrombin complex, fragments were separated by SDS-PAGE, transferred to PVDF membrane and subjected to Edman –degradation as described in the method section. The obtained n-terminal sequences of fragments and their molecular weight is listed.

When TAFI was cleaved by trypsin, which is more potent than thrombin/solulin complex, a somewhat dissimilar fragmentation pattern was generated (Fig 2). SDS-PAGE of the human protein yielded a typical pattern consisting of full length TAFI at 58 kDa, mature TAFIa at 36 kDa, C-terminal processed TAFIa (through cleavage at Arg^330^) at 28 kDa, and the released C-terminal peptide at 8 kDa (Fig 2). Trypsin cleavage occurred at the same site in both the human and bovine protein, creating a 36 kDa TAFIa through truncation at Arg^92 ^(Fig 2). Interestingly, in contrast to human TAFIa, bovine TAFIa was initially proteolytically inactivated by cleavage at the N-terminus, rather than the C-terminus. This processing occurred right after Arg^147^, creating a 29.2 kDa fragment, detected around 32 kDa on SDS gel (Fig 2 and Table 2). The pro-peptide was detected at around 29 kDa (Fig 2). However, it is most likely immediately processed at the C-terminus, as a 25 kDa band was detected that also contained the TAFI N-terminal sequence (Fig 2). Trypsin and small trypsin fragments were also detected, along with the released 6.7 kDa TAFIa N-terminal peptide (Fig 2 and Table 2).

### Bovine TAFIa stability at 37°C

**Figure 3:**
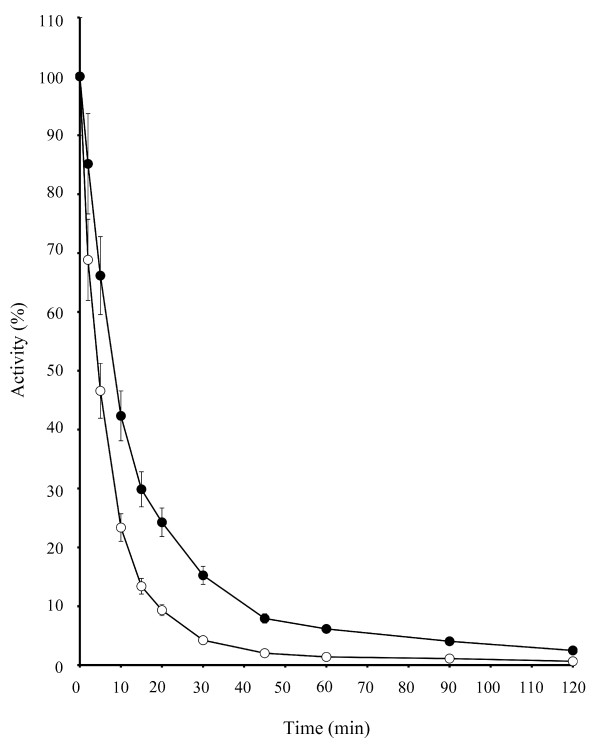
**Bovine TAFIa is more stable at 37°C than human TAFIa**. Bovine (filled circles) or human (open circles) TAFI (3 μg of each) were added thrombin/solulin complex (using optimal conditions as determined in the method section), placed at 37°C, and subjected to HPLC based kinetic assays using Hip-Arg substrate at the indicated intervals. Bovine TAFIa is more stable than human TAFIa, as seen by a half-life that is twice as long (i.e., 10 min vs. 5 min).

The thermal stability of TAFIa was investigated with HPLC based kinetic assays using Hip-Arg as a substrate and the thrombin/solulin complex as an activator (Fig 3). The half-life of bovine TAFIa was longer than that of human TAFIa. Human TAFIa activity decreased by 50% after 5 min, while this decrease in bovine TAFIa activity occurred at 10 min (Fig 3).

### Intrinsic activity of bovine TAFI

**Figure 4:**
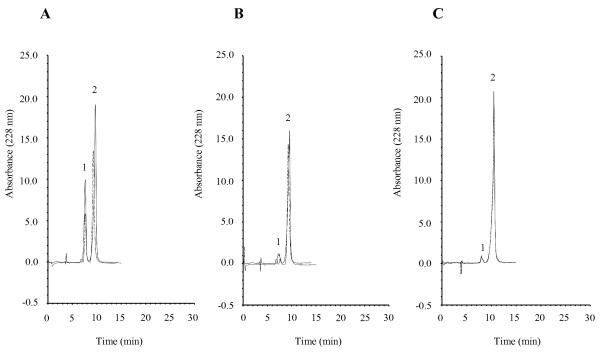
**Bovine TAFI cleaves Hip-Arg substrate**. The intrinsic activity of the bovine and human protein (1 μg) was investigated by incubating TAFI with Hip-Arg substrate in the absence (A) or presence of 5 mM 1, 10-phenantroline (B) or 1 μg TCI (C). The cleaved product, hippuric acid (1), was then separated from the internal standard (2) by RP-HPLC. Bovine TAFI, similar to human TAFI, produces considerable hippuric acid, and this carboxypeptidase activity is abolished by addition of either 1, 10-phenantroline or TCI.

The intrinsic activity of the bovine protein was determined by activity assays using the Hip-Arg substrate and compared to that of human TAFI, by measuring the released hippuric acid by HPLC. TAFI activity was undoubtedly detected using this method (Fig 4A). Moreover, hippuric acid release was blocked when TAFI was incubated with the substrate in the presence of 5 mM 1, 10-phenantroline, a chelating agent and known carboxypeptidase inhibitor (Fig 4B). This confirms that bovine TAFI has genuine intrinsic activity. This activity was also inhibited by 2.3 μM TCI, a potentially physiologically relevant inhibitor of TAFI (Fig 4C).

### Isoelectric point variation between TAFI and TAFIa

Isoelectric focusing revealed that the isoelectric point of TAFI and TAFIa varied greatly in both species (data not shown). The full length bovine protein migrated in the lower end of the pH gradient and appeared as multiple bands between a pI of 6.0 and 6.5, likely due to glycosylation heterogeneity. This migration position was slightly higher than that of the human TAFI, which migrated to a pI of 5.1 to 6.0 and also appeared as multiple isoforms. Upon release of the heavily glycosylated activation peptide, both human and bovine TAFIa appeared as a single band at a much higher pI of around 8.5. Thus, the difference in migration between TAFI in the two species is due to differences in the carbohydrate modifications to their respective pro-peptides.

### Bovine TAFI attenuates clot lysis in vitro

**Figure 5:**
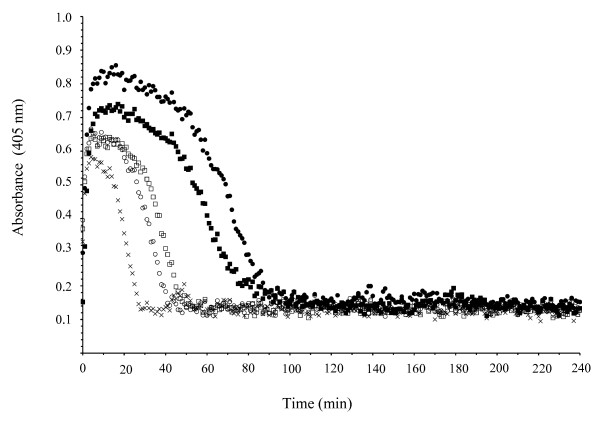
**Bovine TAFIa attenuates clot lysis**. The anti fibrinolytic function of bovine TAFIa was tested and compared to that of human TAFIa in a purified system. Clott formation was initiated by addition of thrombin to fibrinogen in the presence of CaCl_2_. Simultaneously, the dissolution of clot was generated by tPA and plasminogen addition. The change in turbidity was monitored at 405 nm (crosshairs). Upon addition of 1 μg of either bovine TAFI (filled circles) or human TAFI (filled squares), in the presence of Solulin, a delay in clot lysis was observed. A small anti fibrinolytic effect was observed upon addition of 1 μg of bovine TAFI (open circles) and human TAFI (open squares) in the absence of Solulin as well.

The effect of bovine TAFI on fibrinolysis was examined by conducting a fibrinolyses assay in a purified system (Fig 5). The clot was generated in microtiter wells by addition of thrombin to fibrinogen and the clot lysis initiated by further addition of plasminogen and tPA simultaneously. Purified bovine or human TAFI (1 μg) added to the wells, in the presence and absence of solulin, was able to delay clot lysis. Moreover, this effect was reversed by the carboxypeptidase inhibitors, PCI or TCI, confirming capability of bovine (and human) TAFI to effect clot lysis (data not shown).

### Bovine TAFI is a substrate for tissue transglutaminase

**Figure 6:**
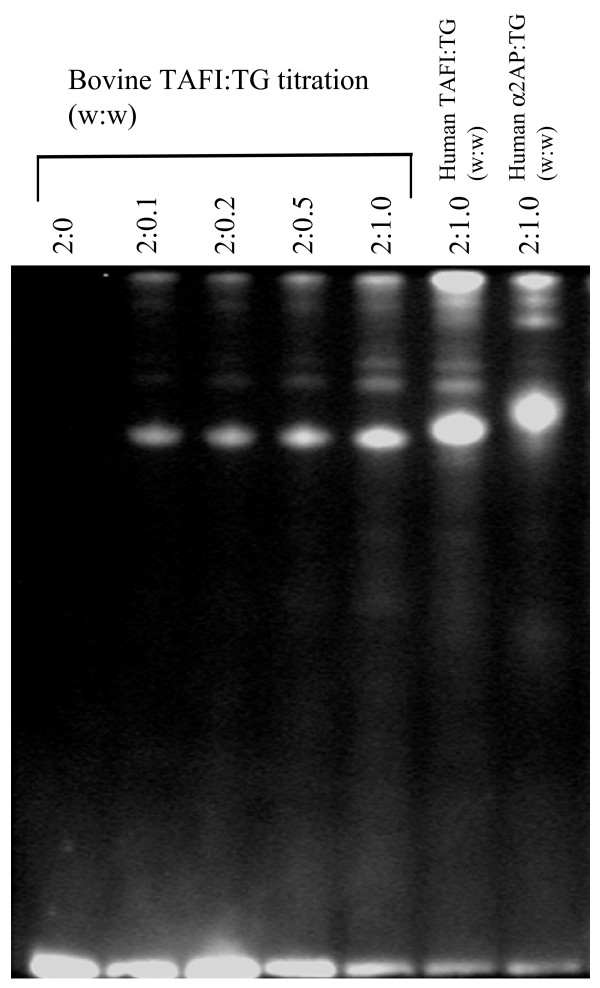
**Bovine TAFI is a substrate for transglutaminases**. Bovine TAFI (2 μg) was incubated with increasing amounts of tissue transglutaminase (TG), in the presence of the fluorescent donor, dansylcadaverine. The reaction products were separated by SDS-PAGE and visualized under UV light. Human TAFI (2 μg) and α2-antiplasmin (2 μg) served as a controls. Note the clear incorporation of dansylcadavarine into bovine TAFI, suggesting that it contains amine acceptor sites and functions as a substrate for transglutaminases.

To test whether bovine TAFI has the potential to become cross-linked to a fibrin meshwork in the same manner as the human protein, we monitored the incorporation of a fluorescent donor, dansylcadaverine, into the protein by tissue transglutaminase. Visualization of SDS-polyacrylamide gels with UV light revealed that both human TAFI and α-2AP (another known tissue transglutaminase substrate) incorporated dansylcadaverine (Fig 6). Importantly, dansylcadaverine was also successfully incorporated into bovine TAFI under these conditions, showing that bovine TAFI can serve as a transglutaminase substrate.

### Bovine TAFI contains four N-linked carbohydrate structures, which are located solely on the activation peptide

**Figure 7:**
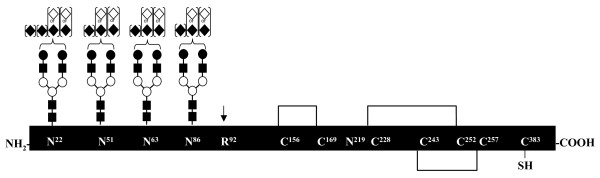
**N-glycans of bovine TAFI**. The site in the N-terminal sequence which is cleaved, releasing the pro-peptide (Arg^92^) and known disulfide bonds of bovine TAFI are shown in the schematic, as well as the glycosylation sites (not drawn to scale). Square brackets indicate that the glycans were observed with and without the particular carbohydrate residue. (Filled squares, N-acetylglucosamine; Open circles, mannose; Filled circles, galactose; Filled diamonds, 5-N-acetylneuraminic acid; Open diamonds, 5-N-glycolylneuraminic acid).

To characterize the glycans of bovine TAFI, we performed tryptic digests with and without subsequent PNGase F treatment, and separated the resulting fragments using RP-HPLC. Fractions containing glycopeptides were purified and analyzed using MALDI-TOF MS and subsequently verified based on their fragmentation using MALDI quadrupole (Q) TOF MS/MS (data not shown). Thirty-five N-glycans were observed from the four N-glycosylation sites present within the N-terminal activation region (i.e., N22, N51, N63, and N86) (Fig 7 and Table 3). Biantennary structures without core fucosylations were the sole structures identified by the glycoanalysis. Hence, the substantial microheterogeneity observed was limited to variations in the contents of the two types of sialic acids, N-glycolylneuraminic acid (Neu5Gc) and N-acetylneuraminic acid (Neu5Ac). Up to four sialic acid residues were observed on the N22 glycans, whereas the rest of the occupied sequons contained a maximum of three sialic acid residues (Fig 7). Peptide mass fingerprinting of tryptic bovine TAFI treated with and without PNGase F revealed that neither of the two potential sites located in the middle of the protein (N219 and N226) was occupied by N-glycans. In contrast, N-linked glycosylation has been detected in human TAFIa. The human protein also exhibits a much greater heterogeneity in the N-linked sugars [18]. This may help account for difficulties in determining the three-dimensional structure of human TAFI zymogen.

**Table 3:** Structure of N-linked carbohydrates in bovine TAFI

**Site**	*N*-Glycans/Occupancy**	**Observed glycopeptide mass (Da)**	**Theoretical glycopeptide mass (Da)**	**Glycan mass (Da)**	Glycans***
**N^22^**	Yes/100%	3157.2	3157.4	1622.6	Gal_2_Man_3_GlcNAc_4_
		3448.3	3448.5	1913.7	Neu5Ac_1_Gal_2_Man_3_GlcNAc_4_
		3464.3	3464.5	1929.7	Neu5Gc_1_Gal_2_Man_3_GlcNAc_4_
		3739.5	3739.6	2204.8	Neu5Ac_2_Gal_2_Man_3_GlcNAc_4_
		3755.5	3755.6	2220.8	Neu5Gc_1_Neu5Ac_1_Gal_2_Man_3_GlcNAc_4_
		4030.6	4030.7	2495.9	Neu5Ac_3_Gal_2_Man_3_GlcNAc_4_
		4046.7	4046.7	2511.9	Neu5Gc_1_Neu5Ac_2_Gal_2_Man_3_GlcNAc_4_
		4062.6	4062.7	2527.9	Neu5Gc_2_Neu5Ac_1_Gal_2_Man_3_GlcNAc_4_
		4321.8	4321.8	2787.0	Neu5Ac_4_Gal_2_Man_3_GlcNAc_4_
		4337.8	4337.8	2803.0	Neu5Gc_1_Neu5Ac_3_Gal_2_Man_3_GlcNAc_4_
		4353.8	4353.8	2819.0	Neu5Gc_2_Neu5Ac_2_Gal_2_Man_3_GlcNAc_4_

**N^51^**	Yes/100%	3533.6	3533.5	1622.6	Gal_2_Man_3_GlcNAc_4_
		3824.7	3824.6	1913.7	Neu5Ac_1_Gal_2_Man_3_GlcNAc_4_
		3840.7	3840.6	1929.7	Neu5Gc_1_Gal_2_Man_3_GlcNAc_4_
		4115.9	4115.7	2204.8	Neu5Ac_2_Gal_2_Man_3_GlcNAc_4_
		4131.9	4131.7	2220.8	Neu5Gc_1_Neu5Ac_1_Gal_2_Man_3_GlcNAc_4_
		4407.1	4406.8	2495.9	Neu5Ac_3_Gal_2_Man_3_GlcNAc_4_
		4423.1	4422.8	2511.9	Neu5Gc_1_Neu5Ac_2_Gal_2_Man_3_GlcNAc_4_
		4439.1	4438.8	2527.9	Neu5Gc_2_Neu5Ac_1_Gal_2_Man_3_GlcNAc_4_

**N^63^**	Yes/100%	2389.9	2390.0	1622.6	Gal_2_Man_3_GlcNAc_4_
		2681.0	2681.1	1913.7	Neu5Ac_1_Gal_2_Man_3_GlcNAc_4_
		2697.0	2697.1	1929.7	Neu5Gc_1_Gal_2_Man_3_GlcNAc_4_
		2972.1	2972.2	2204.8	Neu5Ac_2_Gal_2_Man_3_GlcNAc_4_
		2988.0	2988.2	2220.8	Neu5Gc_1_Neu5Ac_1_Gal_2_Man_3_GlcNAc_4_
		3263.3	3263.3	2495.9	Neu5Ac_3_Gal_2_Man_3_GlcNAc_4_
		3279.2	3279.3	2511.9	Neu5Gc_1_Neu5Ac_2_Gal_2_Man_3_GlcNAc_4_
		3295.3	3295.3	2527.9	Neu5Gc_2_Neu5Ac_1_Gal_2_Man_3_GlcNAc_4_

**N^86^**	Yes/100%	2851.0	2851.2*	1622.6	Gal_2_Man_3_GlcNAc_4_
		3142.1	3142.3*	1913.7	Neu5Ac_1_Gal_2_Man_3_GlcNAc_4_
		3158.1	3158.3*	1929.7	Neu5Gc_1_Gal_2_Man_3_GlcNAc_4_
		3433.2	3433.4*	2204.8	Neu5Ac_2_Gal_2_Man_3_GlcNAc_4_
		3449.2	3449.4*	2220.8	Neu5Gc_1_Neu5Ac_1_Gal_2_Man_3_GlcNAc_4_
		3724.3	3724.5*	2495.9	Neu5Ac_3_Gal_2_Man_3_GlcNAc_4_
		3740.4	3740.5*	2511.9	Neu5Gc_1_Neu5Ac_2_Gal_2_Man_3_GlcNAc_4_
		3756.4	3756.5*	2527.9	Neu5Gc_2_Neu5Ac_1_Gal_2_Man_3_GlcNAc_4_

**N^219^**	unglycosylated				

**N^226^**	unglycosylated				

*Theoretical glycopeptide mass values are based on the transformation of the N-terminal Gln^82 ^to pyroglutamic acid.

**Percent occupancy of N-glycans was determined based on MS experiments in which peptide mass fingerprints of tryptic bovine TAFI treated ± PNGase F were compared to establish the presence of (non/de)-glycosylated peptides (data not shown).

***GlcNAc, N-acetylglucosamine; Man, mannose; Gal, galactose; Neu5Ac, 5-N-acetylneuraminic acid; Neu5Gc, 5-N-glycolylneuraminic acid.

## Discussion

The structure of TAFI and TAFIa/TCI complex has recently been solved using authentic bovine TAFI [42,45]. Here, we present a full biochemical characterization of the bovine protein purified from bovine plasma. The amino acid sequence idenity between the two spieces is 78.6% and all the important sites, such as the catalytic domain, substrate-binding domain, and zinc-binding domain, are fully conserved [45]. Only four of the potential N-linked carbohydrate sites are occupied, all located on the activation peptide. The fifth site, Asn^219^, which is partially glycosylated in the human protein [18], remained unglycosylated in the bovine protein (Fig 7). Accordingly, this site was found to be buried within the protein structure and has previously been suggested to be unglycosylated [42]. Recently, the biochemical importance of human TAFI glycosylation has been studied using TAFI mutants [46]. Interestingly, in some mutants, the absence of carbohydrates increases the activity of full length TAFI, but decreases TAFIa activity. The increase in intrinsic activity is most apparent in the mutants TAFI-N22Q and TAFI-N22Q-N51Q-N63Q. These observations corroborate the finding that, in human TAFI, access to the active site exists [11] and this access site potentially expands upon carbohydrate removal, possibly imparting a catalytic function to sugars [46].

Interestingly, the pronounced microheterogeneity of the TAFI glycans was exclusively generated by the variation in the number and type of sialic acid residues located in the termini of the biantennary complex glycans. Neu5Ac and Neu5Gc were found in the TAFI glycans and both are known to be abundant sialic acids in bovine glycoconjugates. In contrast, humans cannot synthesize Neu5Gc, highlighting a difference between the authentic human and authentic bovine TAFI structure.

Purified bovine TAFI successfully attenuated fibrinolysis of tPA-induced clots in a purified system. Also similar to human TAFI, the bovine protein displays considerable stable intrinsic activity, which can be abolished by the same inhibitors used to inhibit TAFIa. Furthermore, it is most likely crosslinked to the fibrin meshwork during the early stages of fibrinolysis, as the protein seems to act as a substrate for transglutaminases. Bovine TAFI contains potential amine acceptor sites, as evidenced by the successful incorporation of dansylcadavarine into the protein by tissue transglutaminase.

Bovine TAFI, like the human protein, can also be cleaved through proteolysis at Arg^92^, generating the mature form, TAFIa. In contrast to the human TAFI, bovine TAFI is processed into not only the 36 kDa active enzyme, but also a 29 kDa TAFIa fragment following incubation with trypsin. This N-terminally processed TAFIa is missing a 7 kDa N-terminal peptide and is formed through proteolysis of the Arg^147^-Ala^148 ^bond. This cleavage takes place prior to the usual inactivation that occurs at the C-terminus. Human TAFI contains either a Thr or Ala at position 147, depending on the variant. Therefore, an identical N-terminal truncation at this position is not possible [47]. Similar fragmentation has been observed following activation of rat TAFI by plasmin [35]. This may also explain the disordered Lβ2β3 segment observed in the three-dimensional structure of the TAFIa/TCI complex [45].

In human TAFI, substituting His^333 ^with Tyr or Gln increases the half-life of TAFIa for up to 1.5 h, while preserving all characteristics of wild type TAFI [48]. Site-directed mutagenesis of Arg^302^, Arg^320^, and Arg^330 ^produces a molecule much less stable than the wild type protein, suggesting that this instability is concentrated in the 302 – 330 region [21,49]. The naturally occurring mutation of Thr^325 ^to Ile^325 ^has been shown to make human TAFI twice as stable [21,50,51]. Position 325 of the bovine protein is occupied by Ile, which might account for the longer half-life of bovine TAFIa (10 min) compared to the human TAFIa (5 min). Similarly, mutation of Thr^329 ^to Ile^329 ^increases not only the half-life of the cleaved human protein, but also its fibrinolytic effect [21]. Again, this position is occupied by Ile in bovine TAFI.

Substitution of human TAFI residues with corresponding residues of CPB, such as TAFIa-Ile^182^Arg-Ile^183^Glu, does not significantly increase stability. On the contrary, it reduces antifibrinolytic potential. Nevertheless, lower amounts of thrombin-thrombomodulin complex are required in order to generate TAFIa from this mutant [52]. This can explain, at least partly, why lower amounts of proteinases are required to generate bovine TAFIa, in which Lys^182 ^and Glu^183 ^naturally occur in sequence. Indeed, 15 times less solulin/thrombin complex is required to generate bovine TAFIa with activity similar to that of human TAFIa.

In summary, we deduce that human TAFI and bovine TAFI have similar properties. The overall secondary structure is conserved, generation of TAFIa can be achieved in similar manner, and bovine TAFIa produces a measurable effect on fibrinolysis. Thus, the available three-dimensional structure of bovine TAFI is a reliable model for investigation of human TAFI, including its *in vivo *function and the *in vivo *effects of its inhibition.

## Conclusion

The bovine and human TAFI activation occurs at equivalent sites and both TAFIa and TAFI exhibit caroboxypeptidase activity. Additionally, TAFI from both species was found to be substrate for transglutaminases. Minor differences in the enzymatic stability of bovine and human TAFIa was observed as well as differences in the level of glycosylation, isoelectric point and proteolytic by-products in trypsin activation. However, overall the findings suggested that the the two orthologous proteins are similar and that conclusions reached using the bovine TAFI can safely be extrapolated to the human protein.

## Methods

### Materials

Bovine trypsin, 1, 10-phenantroline, phenylmethylsulfonyl fluoride (PMSF), polyethylene glycol 8000 (PEG), and the chromogenic carboxypeptidase substrate, hippuryl-Arg (Hip-Arg), were obtained from Sigma. Ortho-methylhippuric acid and Pefablock SC were from Aldrich. ECH-Lysine Sepharose was from Amersham Biosciences, GE Healthcare (Uppslala, Sweden). Dansylcadaverine was from Molecular Probes (Eugene, OR).

### Proteins

Human TAFI and human α_2_-antiplasmin (α-_2_AP) were purified from normal human plasma (Statens Serum, Institute, Copenhagen, Denmark using plasminogen-depleted plasma and plasminogen-Sepharose affinity chromatography as described previously [4,18]. Guinea pig liver (tissue) transglutaminase (EC 2.3.2.13), human fibrinogen and human thrombin (EC 3.4.21.5) were purchased from Sigma. Recombinanat tPA (EC 3.4.21.68) was purchased from ProSpec-Tany TechnoGene LTD., Rehovot, Israel. Recombinant soluble thrombomodulin (solulin) was a generous gift of Dr. Achim Schuettler (PAION GmbH, Aachen, Germany) and Factor XIIIa from Sanofi-Aventis. Potato carboxypeptidase inhibitor (PCI) and TCI were kind gifts from Prof. Francesc. X. Aviles, Dr. Joan Lopez Arolas, and Dr. Laura Sanglas. TAFI-antiserum was raised commercially (Pel-Freez, Rogers, AR). Human plasminogen was purified by affinity chromatography using ECH-Lysine Sepharose as described previously [53].

### Purification of bovine TAFI

Bovine TAFI was purified essentially as already described [45]. In short, bovine blood (10 L) was collected at the local slaughterhouse and supplemented with 5 mM EDTA to prevent coagulation. The plasma was separated from erythrocytes by centrifugation at 600 × g for 15 min at 22°C. Plasma was incubated with 6% (w/v) PEG, and after 1 h, the precipitated proteins were removed by centrifugation at 10,000 × g for 40 min at 4°C. Plasminogen was removed from the supernatant by affinity chromatography using 1 L of ECH-Lysine Sepharose equilibrated in binding buffer (50 mM NaH_2_PO_4_, pH 7.5 and 100 mM NaCl). Plasminogen-depleted plasma was applied to a 500-ml plasminogen Sepharose column equilibrated in binding buffer, and bovine TAFI was eluted using 50 mM γ-amino-caproic acid. After buffer exchange into 20 mM Tris-Cl (pH 7.5), bovine TAFI was separated from other contaminants by ion-exchange chromatography on a 5-ml HiTrapQ column connected to an AKTA Prime system (Amersham Biosciences, GE Healthcare). The column was eluted, at a flow rate of 1 ml/min, using a 0.5%/min linear gradient of Buffer A (20 mM Tris-Cl, pH 7.5) and Buffer B (20 mM Tris-Cl, pH 7.5 containing 1 M NaCl).

### Polyacrylamide gel electrophoresis

Proteins were separated by SDS-PAGE in 5 – 15% polyacrylamide gels [54]. Samples were boiled for 5 min in the presence of 30 mM dithiothreitol (DTT) and 1% SDS prior to electrophoresis.

### Generation of human and bovine TAFIa

Human and bovine TAFI (1 μg) were incubated with increasing amounts of the thrombin/solulin complex, (0 μg/0 μg to 0.01 μg/0.25 μg) for 30 min at 22°C in 20 mM Tris-HCl and 100 mM NaCl, pH 7.5. For trypsin induced proteolysis, 1 μg TAFI (human and bovine) was incubated with increasing amounts of trypsin (0–0.5 μg) for 20 min at 37°C in 20 mM Tris-HCl and 100 mM NaCl, pH 7.5. All reactions were terminated by addition of Pefablock or PMSF to a final concentration of 5 mM. Optimal conditions to generate TAFIa with peak activity were determined through kinetic assays (using 0.2 μg of TAFI) and SDS-PAGE (using 1.0 μg TAFI) with the physiologically relevant thrombin/solulin complex as an activator only. To activate 1 μg of human TAFI, the optimal trombin/solulin complex ratio (w/w) was 0.06 μg/1.5 μg. Generation of bovine TAFIa was optimal using thrombin/solulin complex ratio of 0.004 μg/0.1 μg to 1 μg TAFI.

### NH_2_-terminal amino acid sequencing

Proteolytic fragments of bovine TAFI generated by trypsin or solulin/thrombin complex were separated by SDS/PAGE. The stacking gel was allowed to polymerize one day prior to electrophoresis, and samples were heated for 3 min at only 80°C prior to separation. After electrophoresis, proteins were transferred to a polyvinylidene difluoride membrane (Immobilon-P, Millipore) in 10 mM CAPS and 10% (v/v) methanol (pH 11) [55]. Alternatively, the TAFI-trypsin or TAFI-solulin/thrombin mixture was applied to an activated ProSorb sample preparation cartridge (Applied Biosystems), according to the manufacturer's instructions. Samples were analyzed by automated Edman degradation using an Applied Biosystems PROCISE™ 494 HT sequencer with on-line HPLC (Applied Biosystems Model 120A) for phenylthiohydantoin analysis.

### Isoelectric focusing

Isoelectric focusing of bovine TAFI was performed essentially as described previously [18]. Briefly, 10 μg of salt-free protein was focused under native conditions in a Ready IEF gel using the MiniProtean III Cell (Biorad) according to the manufacturer's instructions. Bands were focused in a pH gradient of 3 – 10 using 20 mM lysine and 20 mM arginine as a cathode buffer and 7% phosphoric acid as anode buffer (all in H_2_O). Running conditions consisted of 100 V for 60 min, 250 V for 60 min, and 500 V for 30 min. Bands were visualized using IEF staining solution (27% isopropyl alcohol, 10% acetic acid, 0.04% Coomassie Blue R250, and 0.05% Crocein Scarlet in H_2_O).

### HPLC based kinetic activity assay using Hip-Arg substrate

The activity of both full length and mature TAFI was determined essentially as described previously [56]. A 10-μl sample containing 1 μg of bovine TAFI or 0.2 μg TAFIa was incubated with 40 μl of 30 mM Hip-Arg for 40 min. Some samples were incubated for 15 min with 5 mM phenanthroline or 1 μg TCI prior to substrate addition. The reactions were stopped by addition of 50 μl 1 M HCl. Ten microliters of 15 mM ortho-methylhippuric acid was included in the reaction mixture as an internal standard. The reaction products, as well as the internal standard, were extracted using 300 μl ethyl acetate. One-hundred microliters of the extracted sample were lyophilized, solubilized in 100 μl mobile phase buffer [10 mM KH_2_PO_4_, pH 3.4 containing 15% acetonitrile (ACN)], and separated on a reverse phase (RP) HPLC column (PTH C18, 5 μm, 220 × 2.1 mm, Applied Biosystems) using the ÄKTA Ettan system (Amersham Biosciences, GE Healthcare).

### Determination of TAFI kinetic constants

Human and bovine TAFI kinetic properties were essentially determined as described previously, with small modifications [46]. Briefly, 1 μg of the zymogen and 0.1 μg of TAFIa, generated by the thrombin/solulin complex, for both human and bovine protein, were incubated with increasing concentration of the Hip-Arg substrate (0–30 mM), in duplicates, for 60 min at 37°C in a final volume of 60 μl. The reaction was terminated by addition of 20 μl 1 M HCl, neutralized by addition of 20 μl of 1 M NaOH and buffered with 25 μl of 1 M NaH_2_PO_4_, pH 7.4. Upon addition of 60 μl 6% cyanuric chloride dissolved in 1,4-dioxane, the samples were vortexed vigorously and centrifuged at 16000 × g for 5 minutes. The supernatant was subsequently transferred to 96-well microtiter plate and the absorbance was measured at 405 nm in a FLUOStar Omega plate reader (BMG Labtech) using the endpoint mode. The kinetic constants were determined using 4 different graphical methods.

### Thermal stability of TAFIa enzymatic activity

Bovine and human TAFI (3 μg) were mixed with solulin/thrombin complex using the optimal conditions for generation of TAFIa for each species. At the time of reaction termination with pefablock (5 mM final concentration), the reaction mixture was placed at 37°C. At various intervals over 120 min, 0.2 μg of TAFI protein was removed and incubated with Hip-Arg substrate. Kinetic measurements were then performed using HPLC method described above.

### In vitro clot lysis assays

Clot lysis assays were performed essentially as described previously [57] using 96-well microtiter plates, with some modifications. Twenty μl of fibrinogen (20 μl/μg), 1 μl of plasminogen (0.5 μg/μl) and 12.5 μl of factor XIIIa (0.8 μg/μl) were mixed in a final volume of 100 μl in 20 mM Hepes and 150 mM NaCl, 5 mM CaCl_2_, pH 7.4 (reaction buffer) in a set of wells. In a proximate set of wells, 1 μl of tPA (0.002 μg/μl) and 2 μl of thrombin (20 U/ml) were combined in a final volume of 50 μl using the reaction buffer. Clotting was initiated by addition of 50 μl of the fibrinogen/plasminogen/factor XIIIa mixture to wells containing tPA and thrombin. In some wells, 10 μl of solulin (0.1 μg/μl) was added to the tPA/thrombin mixture prior to clot initiation. Purified human or bovine TAFI (1 μg), was added to certain wells containing tPA, thrombin and (+/-) solulin, moments prior to the start of the clotting generation. Some wells contained additionally 1.27 μM TCI or 4.65 μM PCI. The turbidity of the clot was measured continuously at 405 nm in a plate reader (FLUOstar Omega, BMG LABTECH GmbH) at 37°C. The lysis time was defined as the time required for a 50% reduction in optical density.

### Incorporation of dansylcadaverine using tissue transglutaminase

Human TAFI, bovine TAFI, or α-2AP (2 μg of each) were incubated with varying amounts of tissue transglutaminase (0 – 2 μg) for 3 h at 37°C in 20 mM Tris-Cl and 100 mM NaCl (pH 7.5) containing 10 mM Ca^2+^, 0.5 mM DTT, and 0.5 mM dansylcadaverine. The reaction was stopped by addition of 10 mM EDTA, and samples were analyzed by reducing SDS-PAGE. The gel was visualized under UV light.

### Amino acid sequence analysis

To determine the accurate concentration of TAFI used in this study, we performed each analysis in triplicate. For each analysis, approximately 2 μg of purified bovine or human TAFI was dried in 500 μl polypropylene vials. The lids were punctured, and the vials were placed in a 25-ml glass vial equipped with a MinInert valve (Pierce Biotechnology, Rockford, IL, USA). Two-hundred microliters of 6 N HCl containing 0.1% phenol was placed in the bottom of the glass and blown with argon before a vacuum was applied. The samples were incubated at 110°C for 18 h. They were subsequently redissolved in 50 μl 0.20 M sodium citrate loading buffer, pH 2.20 (Biochrom, Cambridge, UK), transferred to microvials, and loaded on a BioChrom 30 amino acid analyzer (Biochrom). Data analysis was performed using software developed in house.

### Proteolytic digestion of bovine TAFI and purification of glycosylated peptides

Modified trypsin (2 μg, Promega, Madison, WI) was added to approximately 40 μg of purified bovine TAFI in 20 mM Tris and 200 mM NaCl (pH 7.5) and then incubated overnight at 37°C. The resulting peptide mixture was split into two samples. N-glycosidase F (1 U, Roche, Mannheim, Germany) was added to one of the samples and incubated overnight at 37°C. The other sample was stored at -18°C. The two samples were applied separately to a reversed phase HPLC column (Jupiter C18 250 mm × 2 mm, 5 μm, 300 Å, Phenomenex, Torrance, CA) connected to an ÄKTA Basic instrument (Amersham Pharmacia Biotech, GE, Uppsala, Sweden). The sample was applied in buffer A [0.06% trifluoroacetic acid (TFA) in water] and eluted using the following three-step gradient in buffer B (0.05% TFA and 90% ACN in water): 5 to 40% in 30 min, 40 to 60% in 5 min, and 60 to 90% in 3 min. Differences in the corresponding chromatograms revealed the fractions potentially containing glycopeptides. These fractions were dried and redissolved in 5% formic acid for further analysis.

### Characterization of glycosylated peptides by matrix-assisted laser desorption/ionization time-of-flight mass spectrometry (MALDI-TOF MS)

The fractions containing glycopeptides were concentrated and desalted using hydrophobic microcolumns packed with Poros R2 (20 μm, Applied Biosystems, Framingham, MA) in GelLoader pipette tips (Eppendorf, Hamburg, Germany) as described elsewhere [58]. The samples were eluted directly onto the MS target with 0.5 μl 2,5-dihydroxybenzoic acid (20 g/L) in 70% ACN and 0.1% TFA. Alternatively, fractions were not desalted and analyzed by mixing 0.5 μl sample and 0.5 μl matrix directly on the target. All samples were analyzed in positive polarity mode by MALDI MS using a Bruker Ultraflex (Bruker Daltonics, Bremen, Germany) with TOF-TOF technology or a MALDI Q-TOF Ultima (Waters, Micromass, Manchester, UK). The spectra were internally calibrated, or external calibration was performed by placing a tryptic lactoglobulin digest near the actual target spot.

## Abbreviations

^1^The abbreviations used are: α-2AP: α2-antiplasmin; ACN, acetonitrile; Gal: galactose; Neu5Gc: 5-N-glycolylneuraminic acid; Neu5Ac: 5-N-acetylneuraminic acid; GlcNAc: N-acetylglucosamine; Hip-Arg: hippuryl-arginine; PMSF: Phenylmethanesulfonyl fluoride; Lys: lysine; Arg: arginine; Man: mannose; MALDI-TOF MS: matrix assisted laser desorption ionization time-of-flight mass spectrometry; PAGE: polyacrylamide gel electrophoresis; PCI: potato carboxypeptidase inhibitor; pI: isolectric point; PVDF: polyvinylidene difluoride; RP-HPLC: reverse phase high performance liquid chromatography; TAFI: zymogen of thrombin activatable fibrinolysis inhibitor; TAFIa: activated form of thrombin activatable fibrinolysis inhibitor; TFA: trifluoroacetic acid; CPB: carboxypeptidase B; tPA: tissue plasminogen activator; TCI: tick carboxypeptidase inhibitor.

## Authors' contributions

ZV performed the majority of the experimental work and wrote the manuscript. MTA and PH performed the carbohydrate analysis. Additionally they provided valuble suggestions and feedback prior to submission of the manuscript. KS and TCH assisted during the purification of the protein. TK cloned the bovine TAFI cDNA and provided the sequence. JJE supervised the experimental work, revised and finalized the manuscript. All authors read and approved the final manuscript.
